# Effect of a low glycaemic index diet during pregnancy on maternal and cord blood metabolomic profiles: results from the ROLO randomized controlled trial

**DOI:** 10.1186/s12986-019-0378-z

**Published:** 2019-08-27

**Authors:** Linda Marchioro, Aisling A. Geraghty, Olaf Uhl, Engy Shokry, Eileen C. O’Brien, Berthold Koletzko, Fionnuala M. McAuliffe

**Affiliations:** 1 Division of Metabolic and Nutritional Medicine, Department of Paediatrics, Dr. von Hauner Children’s Hospital, University hospital, LMU Munich, Lindwurmstraße 4, D-80337 Munich, Germany; 2 UCD Perinatal Research Centre, School of Medicine, University College Dublin, National Maternity Hospital, Dublin, Ireland

**Keywords:** Glycaemic index, Macrosomia, Pregnancy, Dietary intervention, Metabolomics, Maternal blood, Cord blood, Lipid metabolism, Fatty acid metabolism

## Abstract

**Background:**

Elevated post-prandial blood glucose during pregnancy has been associated with adverse pregnancy and offspring outcomes, such as maternal gestational diabetes and excessive foetal growth. The ROLO Study is a randomized controlled trial (RCT) investigating the effect of a low glycaemic index (GI) diet in pregnancy to prevent foetal macrosomia (birth weight > 4000 g). We described the impact of a low-GI diet on the maternal and feto-placental unit metabolism by studying how the ROLO intervention affected maternal and cord blood metabolomes.

**Methods:**

Fasting maternal plasma samples pre- and post-intervention of 51 pregnant women and 132 cord blood samples were measured with a targeted metabolomics approach using liquid-chromatography coupled to tandem mass spectrometry. The differences between RCT groups were explored via multivariate models with covariates correction. Significance was set at Bonferroni-corrected level of 0.05.

**Results:**

A total of 262 metabolites species, sums and ratios were investigated. While no metabolite reached statistical significance after Bonferroni correction, many maternal phospholipids and acylcarnitines were elevated in the intervention group at uncorrected 0.05 alpha level. Most species contained saturated and monounsaturated fatty acid chains with 16 or 18 carbon atoms. In cord blood, no differences were identified between RCT groups.

**Conclusions:**

A low-GI diet in pregnancy was associated with a trend to modest but consistent changes in maternal lipid and fatty acid metabolism. The intervention seemed not to affect foetal metabolism. Our exploratory findings may be used to direct further investigations about low GI diets before and during pregnancy, to improve patient care for pre-conceptional and pregnant women with lipid dysregulations and potentially modulate the offspring’s risk for future metabolic diseases.

**Trial registration:**

Current Controlled Trials ISRCTN54392969.

**Electronic supplementary material:**

The online version of this article (10.1186/s12986-019-0378-z) contains supplementary material, which is available to authorized users.

## Background

Pregnancy is a period of major endocrine and metabolic changes which modulate both maternal and child’s health [10]. Pregnancy exposures such as gestational diabetes mellitus (GDM), elevated maternal pre-pregnancy body-mass-index (BMI) and gestational weight gain (GWG) are risk factors for type 2 diabetes, overweight, and metabolic syndrome not only in the mother [3, 10, 27] but also in the offspring, as suggested by the numerous indications for the Developmental Origins of Health and Disease (DOHaD) hypothesis [12, 24]. Therefore, it is important to understand the mechanisms driving these changes and to build the foundation for acting timely to prevent the onset of disease in mothers and children.

One possible intervention strategy involves targeting maternal blood glucose levels. Elevated fasting and post-prandial blood glucose levels, even in absence of overt pre-existing diabetes or GDM, have been associated with adverse outcomes for mother and child [33]. Nutritional and dietary measures to ameliorate glycaemic control are standard in pregnant women with diabetes and GDM [21]. One dietary parameter of interest is the glycaemic index (GI). The GI of a carbohydrate-containing food, expressed on a scale from 0 to 100, quantifies the peak in the blood sugar concentrations after ingestion of the food [25]; therefore, the consumption of low GI foods is considered desirable to achieve good glycaemic control.

Several randomized control trials to investigate the effect of low-GI diet on maternal and new-born outcomes have been conducted, reporting favourable effects on maternal glycaemic control but heterogeneous results regarding offspring outcomes [48]. In particular, there is a lack of knowledge regarding how a low-GI diet may impact maternal and feto-placental metabolism at a molecular level in a real-environment clinical setting. In this study, we aim to provide insights into this question using a metabolomics approach. Metabolomics is the omics branch investigating small (< 1.5 kDa) intermediates and products of metabolic reactions and is an established tool in metabolism research, with potential applications in precision medicine and personalized patient care [2, 23, 37]. Ultimately, this exploratory study could inform clinical practise on treatments for pregnant women, aimed at increasing maternal wellbeing and decreasing the offspring’s risk for future metabolic conditions.

## Materials and methods

### Study participants and data collection

This was a secondary analysis conducted on data from the ROLO study. The ROLO study (Randomised cOntrol trial of LOw glycaemic index diet versus no dietary intervention to prevent recurrence of foetal macrosomia, 2007–2011, Dublin, Ireland) tested the hypothesis of a low-GI diet in pregnant women to reduce birth weight in secundigravida with a previous macrosomic child (birth weight > 4000 g); the intervention group (*n* = 394) received an educational session about low-GI diet at the beginning of the second trimester, while the standard group (*n* = 406) received standard care only (trial registration: Current Controlled Trials ISRCTN54392969) [46].

Recruitment and the first study visit took place at the end of the first pregnancy trimester (median: 13th gestation week) and rapidly followed by the educational session (median: 15th week); additional visits were held at 28th and 34th weeks of gestation.

Maternal age at delivery, early pregnancy weight and BMI, weight at 34th week, gestational age at delivery, newborn’s sex, weight and length were documented. Gestational weight gain (GWG) was defined as weight at last measured visit (38th or 40th gestational week) after subtraction of early pregnancy weight; for cases with missing weight at 38th or 40th week, GWG was imputed by adding the overall ROLO median GWG between 34th and 38th week to the weight measured at 34th week. Newborn’s ponderal index at birth was calculated as 100 ∙ birth weight (g) / birth length^3^ (cm^3^).

Maternal fasting blood samples were collected at recruitment and again at the 28th week. Cord blood was collected at delivery. Total, HDL and LDL cholesterol were measured via Roche cholesterol oxidase method and direct HDL Roche 3rd generation method, respectively, on the cobas C702 module of the Roche Cobas 8000 analyser (Roche Diagnostics GmbH, Penzberg, Germany); the Friedewald equation was used to estimate LDL-cholesterol concentrations [7].

Three-days food diaries were collected in each pregnancy trimester and evaluated by a research dietitian via WISP software version 3.0 (Tinuviel Software, Llanfechell, UK) [32]. From these data, the absolute GI intake and the proportion of energy derived from saturated, monounsaturated and polyunsaturated fat intake, expressed as percentage of total energy intake (% kcal), were derived.

### Metabolomics measurements

For subgroups of evaluable mother/child pairs, aliquots of the collected samples were provided for metabolomics analysis. Plasma samples were measured in a targeted approach using liquid chromatography coupled to tandem mass spectrometry (LC/MS-MS) in the laboratory of the Division of Metabolic and Nutritional Medicine, Dr. von Hauner Children’s Hospital (LMU Munich). Five classes of metabolites were analysed: amino acids (AA), non-esterified fatty acids (NEFA), acylcarnitines (AC), branched chain keto acids (BCKA) and intermediates of TCA cycle (TCA), and phospholipids (PL) (including sphingomyelins (SM), diacyl-phosphatidylcholines (PCaa), acyl-alkyl-phosphatidylcholines (PCae) and lysophosphatidylcholines (LPC)). After preparation, samples were randomly distributed in 4 96-wells batches with maternal blood (1–4) and 3 batches with cord blood (5–7). In each batch, up to 80 test samples were measured together with 6 quality control (QC) samples (prepared as pooled mixture of the samples from batch 1, for maternal blood, or from batch 5, for cord blood) and 10 standards used for quantification. The injection of the samples was randomized in each run, with QC and standards being injected regularly every 6–7 test samples. Measurements and QC were performed separately for each blood source.

#### Samples preparation

Proteins of 50 μL plasma were precipitated on a plate with PTFE filter elements by adding 450 μL methanol including internal standards (ISD). After centrifugation the filtrate was split into aliquots for the analyses of individual methods.

#### Amino acids

Fifty μL of the filtrate was used for the derivatization to AA butyl ester with hydrochloric acid in 1-butanol according to the method described by Harder et al. [15]. A set of labeled amino acid standards (set A, Cambridge Isotope Laboratories) mixed with L-Asparagine (15 N2, 98%, Cambridge Isotope Laboratories) and L-Tryptophan (Indole-D5, 98%, Cambridge Isotope Laboratories) was used as internal standard (ISD). After evaporation, the residues were dissolved in water/methanol (80:20) with 0.1% formic acid and determined by LC-MS/MS equipped with 150 × 2.1 mm, 3.5 μm particle size C18 HPLC column (X-Bridge, Waters, Milford, USA) and 0.1% heptafluorobutyric acid as ion pair reagent in mobile phase A (water) and B (methanol). MS detection was performed with a triple quadrupole mass spectrometer (API2000, Sciex, Darmstadt, Germany) with atmospheric pressure chemical ionization source (APCI) operating in positive ion ionization mode.

#### NEFA

Fifty μL of the filtrate was diluted with100 μL methanol and injected to a LC-MS/MS operating in negative electrospray ionization (ESI) mode for identification of NEFA as described by Hellmuth et al. [16]. Uniformly 13C-labeled palmitic acid was used as ISD. Samples were injected to an HPLC system (1200, Agilent, Waldbronn, Germany) with a UPLC diphenyl column (Pursuit UPS Diphenyl, Agilent, Waldbronn, Germany). Five mM ammonium acetate and 2.1 mM acetic acid in water were used as mobile phase A and acetonitrile/isopropanol (80/20) as mobile phase B. A hybrid triple quadrupole mass spectrometer (4000 QTRAP, Sciex, Darmstadt, Germany) operating in negative ESI multiple reaction monitoring mode (MRM) mode was used for MS detection. This method allows for the separation of NEFA species differing in chain length and number of double bonds, but not in the position of double bonds. The analytical process was post-processed using Analyst software version 1.6.2.

#### BCKA and TCA

Organic and keto-acids were measured by a modified method based on previously published procedures [4, 30]. D3-methylmalonic acid (Cambridge Isotope Laboratories, Teweeksbury, MA, USA) was used as ISD. One hundred μL of the supernatant were evaporated to dryness and re-suspended in 50 μL water. Five μL of the extracted samples were injected by HPLC system (1200, Agilent, Waldbronn, Germany) on a Kinetex F5 core-shell HPLC column, 150 × 2.1 mm, 2.6 μm particle size (Kinetex F5, Phenomenex, Aschaffenburg, Germany) for chromatographic separation of molecular species. The mobile phase A was water with 1% formic acid and mobile phase B was composed of methanol/isopropanol (50/50) with 1% formic acid. A gradient elution at a flow rate of 250 μL/min was held constant for 1 min with 1% B, raised to 65% B within 6 min, and turned back to initial conditions of 1%B within 0.5 min. The triple quadrupole mass spectrometer (4000QTRAP, Sciex, Darmstadt, Germany) was operated in negative scheduled MRM mode using ESI.

#### Phospholipids

Phospholipids were analyzed as described by Uhl et al. [45] using LPC (13:0) and PC (14:0/14:0) (Avanti Polar Lipids, Alabaster, Alabama, USA) as ISD. Thirty μL of the centrifuged supernatant were mixed for 20 min at 600 rpm with 500 μl methanol containing 1.2 mM ammonium acetate. Phospholipids were analyzed by flow-injection analysis (FIA) in a triple quadrupole mass spectrometer (QTRAP4000, Sciex, Darmstadt, Germany) coupled to a LC system (1200 Agilent, Waldbronn, Germany). ESI was used in positive ionization mode. MS/MS analysis was run in positive MRM mode with 184 Da (choline head group) as product ion for the PL. Analyst 1.6.2 software, followed by in-house processing with the statistical software R [44], was used for post-processing. The number of carbon atoms (XX) and double bonds (Y) is expressed in the form C XX:Y.

#### Acylcarnitines

D3-carnitine-C2, D3-carnitine-C8 and D3-carnitine-C16 (all Cambridge Isotope Laboratories, Teweeksbury, MA, USA) were used as ISDs. FIA with isocratic elution with 76% isopropanol, 19% methanol and 5% water was used to measure acylcarnitines. The mass spectrometer (4000 QTRAP, Sciex, Darmstadt, Germany) was equipped with ESI and operated in positive ionization mode.

#### Quality control

To ensure precision of the measured samples, 6 QC samples, pooled from the test samples, were measured in each batch. Batches with a coefficient of variation (CV) > 25% were excluded. If at least 75% of the batches for a metabolite passed the intra-batch quality control, the inter-batch CV was calculated, and the metabolite was kept if CV < 30%. In each batch, at most one QC sample was allowed to be an outlier (defined as measurement further away than 1.5 interquartile range (IQR) from the next measurement) and removed.

After quality control, 6 sums and ratios were additionally calculated: sums of PCaa, PCae, total PC, total SM, ratio of total SM to total PC, ratios of NEFA 18:1/18:0 and 16:1/16:0 depicting SCD-1 activity [6], and five ratios of AC 2:0 to mid-chain AC (AC 14:0, 16:0, 16:1, 18:0, 18:1) depicting fatty acid oxidation (FAO) [29].

### Statistical treatment

#### Data preparation

QC and statistical treatment of the data were performed using the statistical software R version 3.4.3 [44].

To ensure interpretability of the results, only subjects with covariates information, mothers with longitudinal metabolomics data (full set analysis) and babies born after the 37th gestational weeks were included. The final sample sizes for maternal and cord analyses were thus 51 and 132 subjects, respectively. Metabolomics outliers identification and removal was performed before models calculation within each blood source and visit time point; outliers were defined as concentration values further away than 3 standard deviations from the next measurements.

Covariables are presented descriptively as median (IQR) or as absolute number (percentage), stratified by blood source and RCT arms. Variables were compared in the two RCT arms using Mann Whitney U-tests.

#### Main models

For each metabolite, a generalized additive model (GAM) was calculated using the function gam() from the R package mgcv [47]. In the following notations, s(∙) indicates a non-linear effect and 1|∙ the random intercept.

The models for maternal metabolites were calculated as follows: metabolite at 28 weeks ~ RCT group + maternal BMI + metabolite at 13 weeks + s (sample storage time) + 1|batch number. Full results are presented in Additional file 1. Maternal age was included in a first step, but since preliminary results showed weak to no associations with maternal age, the variable was removed to preserve statistical power. For some metabolites of interest, a sensitivity analysis was conducted by re-calculating the models after trimming the highest and lowest 5 concentration values. Additional univariate and multivariate sensitivity analyses (including the association of selected metabolites with dietary fat intakes) and their results are presented in Additional file 2.

The models for cord metabolites were calculated as follows: metabolite ~ RCT group + maternal BMI + gestational age + foetal sex + s (sample storage time) + 1|batch number. As sensitivity analysis, the following covariates were included one at a time in the model: ponderal index (PI) of the new-born, maternal GWG, cord HDL, LDL and total cholesterol. Since the results did not substantially change, these are not presented. Additionally, the calculation of the main model was repeated by including only those maternal/child dyads for which also maternal blood was analysed.

#### Significance and reported values

From these models, the standardized beta estimates, uncorrected and Bonferroni-corrected *p*-values and 95% confidence interval of the beta estimate for the RCT variable are reported. Associations with Bonferroni-corrected p-values < 0.05 were defined as ‘significant’, associations with uncorrected p-values < 0.05 were defined as ‘trends’. False discovery rate (FDR) p-values correction was also applied, but, since the significant metabolites did not differ between the two approaches, we used only Bonferroni due to its easier interpretation. Metabolites with uncorrected RCT p-value < 0.05 were visually inspected via grouped boxplots. Results of these models are presented in graphical form via Manhattan plots.

## Results

### Covariates

The covariates, stratified for the two subpopulations (maternal blood samples/cord blood samples), are presented in Table 1. Maternal samples from 51 mothers (control/intervention: 26/25) and 132 cord blood samples (68/64) were included in the analysis; both maternal and cord blood were analysed for 48 mother/child dyads. Only GI in trimester 2 (i.e. after the intervention) and gestational age were significantly different between the RCT arms.

**Table 1:** Demographic and clinical variables of the subjects included in the analyses

	Maternal blood			Cord blood		
	Control group (*n* = 26)	Intervention group (*n* = 25)	*p*-value	Control group (*n* = 68)	Intervention group (*n* = 64)	*p*-value
Maternal anthropometry and blood parameters						
Maternal BMI (kg/m^2^)	24.34 ± 4.40	26.36 ± 5.33 [1]	NS	25.38 ± 4.27	26.24 ± 4.36 [1]	NS
Maternal age (years)	33.37 ± 5.73	32.30 ± 6.89	NS	33.00 ± 5.04	32.45 ± 6.31	NS
Gestational weight gain (kg)	13.20 ± 4.06 [7]	12.18 ± 4.74 [7]	NS	13.20 ± 4.59 [13]	12.40 ± 4.69 [16]	NS
Fasting glucose, 13th week (mmol/l)	4.50 ± 0.40 [1]	4.40 ± 0.50	NS	4.50 ± 0.40 [4]	4.50 ± 0.40 [2]	NS
Fasting glucose, 28th week (mmol/l)	4.60 ± 0.42 [2]	4.50 ± 0.30	NS	4.50 ± 0.60 [3]	4.50 ± 0.50 [2]	NS
Total cholesterol, 13th week (mmol/l)	4.77 ± 0.92 [9]	4.81 ± 1.01 [8]	NS	4.76 ± 1.06 [28]	5.25 ± 1.32 [25]	0.072
Total cholesterol, 28th week (mmol/l)	6.31 ± 1.30 [8]	6.39 ± 1.06 [9]	NS	6.16 ± 1.13 [26]	6.46 ± 1.30 [24]	NS
HDL cholesterol, 13th week (mmol/l)	0.80 ± 0.26 [9]	0.93 ± 0.38 [8]	NS	0.78 ± 0.28 [28]	0.87 ± 0.40 [25]	NS
HDL cholesterol, 28th week (mmol/l)	0.97 ± 0.32 [8]	1.04 ± 0.34 [9]	NS	0.93 ± 0.39 [26]	1.02 ± 0.40 [24]	NS
LDL cholesterol, 13th week (mmol/l)	3.35 ± 0.64 [9]	3.45 ± 1.03 [8]	NS	3.34 ± 0.89 [28]	3.64 ± 0.90 [25]	NS
LDL cholesterol, 28th week (mmol/l)	4.71 ± 1.40 [8]	4.62 ± 1.22 [9]	NS	4.26 ± 1.33 [26]	4.75 ± 1.23 [24]	NS
LDL cholesterol, difference 28th - 13th week (mmol/l)	0.83 ± 1.47 [9]	1.06 ± 0.60 [9]	NS	0.98 ± 1.17 [29]	1.01 ± 0.83 [27]	NS
Maternal diet						
Daily energy intake T1 (kcal)	1803.28 ± 357.75 [4]	1873.09 ± 420.48 [5]	NS	1876.03 ± 506.89 [10]	1793.15 ± 467.24 [13]	NS
Daily energy intake T2 (kcal)	1839.30 ± 279.74 [4]	1775.72 ± 532.19 [4]	NS	1932.25 ± 434.53 [10]	1759.49 ± 534.65 [11]	0.085
Daily energy intake T3 (kcal)	1915.07 ± 334.97 [4]	1800.89 ± 450.74 [3]	NS	2022.50 ± 494.67 [10]	1768.26 ± 473.59 [10]	0.027
Daily GI T1	57.10 ± 6.76 [4]	57.09 ± 3.57 [6]	NS	57.13 ± 5.45 [11]	56.98 ± 4.38 [14]	NS
Daily GI T2	58.52 ± 2.99 [4]	55.25 ± 2.95 [6]	0.004	57.66 ± 3.93 [11]	55.65 ± 3.42 [14]	0.001
Daily GI T3	58.60 ± 3.97 [4]	56.61 ± 3.72 [6]	NS	57.76 ± 4.83 [11]	56.15 ± 4.21 [14]	NS
Total fat intake T1 (g)	71.35 ± 22.41 [4]	70.32 ± 26.29 [5]	NS	76.10 ± 22.08 [10]	72.13 ± 25.26 [13]	NS
Saturated fat intake T1 (g)	30.26 ± 11.38 [4]	27.74 ± 11.65 [5]	NS	31.12 ± 10.23 [10]	27.35 ± 14.41 [13]	0.057
Monounsaturated fat intake T1 (g)	22.59 ± 7.08 [4]	23.26 ± 6.53 [5]	NS	23.81 ± 6.42 [10]	23.91 ± 7.22 [13]	NS
Polyunsaturated fat intake T1 (g)	10.87 ± 6.05 [4]	10.73 ± 5.37 [5]	NS	10.87 ± 5.65 [10]	10.80 ± 5.11 [13]	NS
Total fat intake T2 (g)	71.80 ± 13.20 [4]	70.49 ± 26.74 [4]	NS	75.01 ± 24.54 [10]	70.25 ± 25.53 [11]	0.077
Saturated fat intake T2 (g)	26.97 ± 10.95 [4]	24.51 ± 8.20 [4]	NS	29.56 ± 10.96 [10]	24.51 ± 11.56 [11]	0.019
Monounsaturated fat intake T2 (g)	21.91 ± 4.80 [4]	22.75 ± 7.53 [4]	NS	23.34 ± 8.38 [10]	22.63 ± 8.73 [11]	NS
Polyunsaturated fat intake T2 (g)	11.39 ± 5.60 [4]	10.79 ± 6.03 [4]	NS	12.23 ± 5.41 [10]	10.55 ± 4.76 [11]	NS
Total fat intake T3 (g)	75.28 ± 22.81 [4]	73.48 ± 30.10 [3]	NS	79.31 ± 22.91 [10]	70.82 ± 29.12 [10]	NS
Saturated fat intake T3 (g)	28.49 ± 10.77 [4]	29.32 ± 18.93 [3]	NS	29.92 ± 12.11 [10]	26.37 ± 13.87 [10]	NS
Monounsaturated fat intake T3 (g)	23.33 ± 6.30 [4]	24.43 ± 8.99 [3]	NS	25.56 ± 7.54 [10]	21.56 ± 10.81 [10]	NS
Polyunsaturated fat intake T3 (g)	10.17 ± 5.23 [4]	13.01 ± 5.69 [3]	NS	12.04 ± 5.32 [10]	11.34 ± 5.36 [10]	NS
Total fat intake T1 (%kcal)	0.37 ± 0.09 [4]	0.34 ± 0.06 [5]	NS	0.38 ± 0.07 [10]	0.35 ± 0.08 [13]	NS
Saturated fat intake T1 (%kcal)	14.76 ± 5.36 [4]	13.49 ± 4.31 [5]	NS	15.16 ± 4.63 [10]	13.55 ± 4.09 [13]	NS
Monounsaturated fat intake T1 (%kcal)	11.68 ± 3.59 [4]	11.20 ± 2.93 [5]	NS	11.76 ± 3.14 [10]	11.34 ± 3.75 [13]	NS
Polyunsaturated fat intake T1 (%kcal)	5.71 ± 2.18 [4]	5.31 ± 1.74 [5]	NS	5.64 ± 1.92 [10]	5.09 ± 2.33 [13]	NS
Total fat intake T2 (%kcal)	0.36 ± 0.06 [4]	0.36 ± 0.06 [4]	NS	0.36 ± 0.07 [10]	0.35 ± 0.06 [11]	NS
Saturated fat intake T2 (%kcal)	13.39 ± 3.91 [4]	12.93 ± 4.72 [4]	NS	13.60 ± 3.87 [10]	12.56 ± 4.36 [11]	NS
Monounsaturated fat intake T2 (%kcal)	11.21 ± 2.81 [4]	10.76 ± 2.78 [4]	NS	11.57 ± 2.56 [10]	10.90 ± 3.14 [11]	NS
Polyunsaturated fat intake T2 (%kcal)	5.63 ± 1.86 [4]	5.93 ± 1.86 [4]	NS	5.57 ± 1.91 [10]	5.48 ± 1.97 [11]	NS
Total fat intake T3 (%kcal)	0.36 ± 0.06 [4]	0.36 ± 0.04 [3]	NS	0.36 ± 0.06 [10]	0.36 ± 0.06 [10]	NS
Saturated fat intake T3 (%kcal)	13.50 ± 4.34 [4]	14.10 ± 4.27 [3]	NS	13.73 ± 4.00 [10]	13.24 ± 3.76 [10]	NS
Monounsaturated fat intake T3 (%kcal)	11.36 ± 2.62 [4]	11.60 ± 2.67 [3]	NS	11.39 ± 2.07 [10]	11.33 ± 2.72 [10]	NS
Polyunsaturated fat intake T3 (%kcal)	5.52 ± 1.91 [4]	5.93 ± 1.61 [3]	NS	5.43 ± 2.07 [10]	5.59 ± 1.63 [10]	NS
New-born anthropometry						
Gestational age (days)	280 ± 10.00 [1]	284 ± 8.00	0.022	282 ± 12.25	284 ± 10.00	0.048
Child sex - female	17 (65%)	11 (44%)	NS	39 (57%)	30 (47%)	NS
Birth weight (g)	3860 ± 460.00	4090 ± 480.00	NS	3995 ± 532.50	4105 ± 550.00	NS
Birth length (cm)	52.00 ± 3.00 [6]	52.75 ± 1.88 [7]	NS	52.50 ± 2.75 [13]	53.00 ± 3.00 [15]	NS
Birth ponderal index (100 g/cm^3^)	2.60 ± 0.28 [6]	2.81 ± 0.50 [7]	NS	2.73 ± 0.44 [13]	2.70 ± 0.41 [15]	NS

Values are reported in absolute number (%) or median ± interquartile range. Numbers in square brackets indicate the amount of missing values. *P*-values were calculated via Mann-Whitney U-tests and chi-square tests. *Abbreviations: GI* glycaemic index, *T1/T2/T3* (Pregnancy) Trimester 1/2/3, *NS* non significant

### Metabolites

Two hundred twenty-nine analytes were used in the analyses for maternal blood, 197 in cord blood. A total of 257 analytes passed the quality control in at least one of the blood sources, 170 of which in both. These were: sum of hexoses (H1), 22 AA, 33 NEFA, 26 AC (including free carnitine), 8 TCA, 2 BCKA, 7 LPC, 24 PCaa, 26 PCae, and 21 SM. In both blood sources it was additionally possible to investigate sums and ratios.

#### RCT and maternal blood

**Fig. 1:**
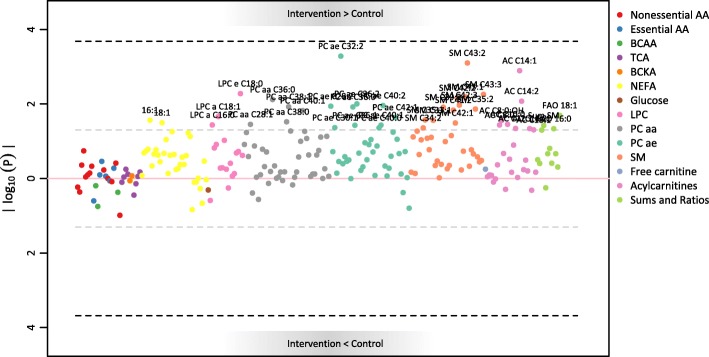
Manhattan plot for the association of maternal metabolites with the RCT arm. Associations were calculated via generalized additive model with correction for maternal BMI, baseline metabolite levels, sample storage time (non-linear effect) and random intercept for batch number. The full list of legend abbreviations is available in the methods section

After Bonferroni correction, no significant differences were found (see Fig. 1 and Additional file 1). However, 40 metabolites were higher in intervention than control arm at uncorrected 0.05 level (see Table 2): two NEFA (16:1 and 18:1), eight mid-chain AC (with chain length from 8 to 18 carbon atoms), three LPC (with chain lengths of 16 and 18 carbon atoms), 15 PCaa and PCae (13 of which containing a 16- or 18-carbon atom saturated or monounsaturated fatty acid (FA) chain), and 12 SM (with saturated or monounsaturated FA chains). The sum of SM and two FAO markers were also significantly higher (uncorrected 0.05 level) in the intervention group; no other sum or ratio was different between the groups.

**Table 2:** Results from maternal blood analysis. Since no metabolite was significant after Bonferroni correction, only results with uncorrected *p* < 0.05 are presented here (for complete results, including Bonferroni correction: see Additional file 2). Beta > 0 indicates higher values of the analyte in the intervention group

Analyte	Analyte group	Beta (Std.)	95% CI (Std.)	*p*-value
16:1	NEFA	0.61	(0.09, 1.12)	0.027
18:1	NEFA	0.62	(0.07, 1.17)	0.032
AC C8:0	Acylcarnitines	0.58	(0.05, 1.11)	0.037
AC C8:0:OH	Acylcarnitines	0.59	(0.09, 1.1)	0.028
AC C10:0	Acylcarnitines	0.6	(0.06, 1.14)	0.035
AC C12:1	Acylcarnitines	0.57	(0.03, 1.11)	0.045
AC C14:1	Acylcarnitines	0.87	(0.38, 1.37)	0.001
AC C14:2	Acylcarnitines	0.73	(0.21, 1.25)	0.008
AC C16:2	Acylcarnitines	0.51	(0.03, 1)	0.046
AC C18:1	Acylcarnitines	0.57	(0.02, 1.12)	0.049
LPC a C16:0	LPC	0.52	(0.05, 0.98)	0.037
LPC a C18:1	LPC	0.62	(0.11, 1.13)	0.022
LPC e C18:0	LPC	0.65	(0.22, 1.07)	0.005
PC aa C28:1	PC aa	0.48	(0.05, 0.92)	0.035
PC aa C36:0	PC aa	0.64	(0.19, 1.09)	0.008
PC aa C38:0	PC aa	0.54	(0.07, 1.02)	0.03
PC aa C38:1	PC aa	0.65	(0.17, 1.13)	0.012
PC aa C40:1	PC aa	0.55	(0.13, 0.97)	0.015
PC ae C28:2	PC ae	0.58	(0.15, 1.01)	0.011
PC ae C30:1	PC ae	0.53	(0.03, 1.02)	0.043
PC ae C32:2	PC ae	0.77	(0.37, 1.16)	5.21e-04
PC ae C36:0	PC ae	0.63	(0.16, 1.1)	0.012
PC ae C36:1	PC ae	0.54	(0.05, 1.04)	0.037
PC ae C36:2	PC ae	0.73	(0.2, 1.26)	0.01
PC ae C40:0	PC ae	0.53	(0.03, 1.03)	0.044
PC ae C40:1	PC ae	0.55	(0.05, 1.05)	0.037
PC ae C40:2	PC ae	0.51	(0.13, 0.88)	0.011
PC ae C42:1	PC ae	0.47	(0.08, 0.86)	0.023
SM C34:2	SM	0.53	(0.03, 1.03)	0.044
SM C35:1	SM	0.58	(0.08, 1.07)	0.027
SM C35:2	SM	0.61	(0.15, 1.06)	0.014
SM C37:1	SM	0.61	(0.09, 1.13)	0.027
SM C39:2	SM	0.59	(0.15, 1.02)	0.012
SM C41:2	SM	0.58	(0.14, 1.03)	0.015
SM C42:1	SM	0.52	(0.06, 0.98)	0.032
SM C42:2	SM	0.71	(0.22, 1.19)	0.007
SM C42:3	SM	0.62	(0.16, 1.07)	0.011
SM C43:1	SM	0.74	(0.23, 1.24)	0.006
SM C43:2	SM	0.84	(0.38, 1.29)	7.94e-04
SM C43:3	SM	0.68	(0.23, 1.14)	0.006
Sum SM	Sums and Ratios	0.55	(0.05, 1.06)	0.038
FAO 16:0	Sums and Ratios	0.52	(0.02, 1.02)	0.046
FAO 18:1	Sums and Ratios	0.62	(0.11, 1.12)	0.02

*Abbreviations*: *CI* Confidence interval, *Std*. Standardized estimate, *NEFA* Non-esterified fatty acid, *AC* Acylcarnitine, *LPC a* Lysophosphatidylcholine with acyl bond, *PC aa* diacyl-phosphatidylcholine, *PC ae* acyl-alkyl-phosphatidylcholine, *SM* Sphingomyelin, *FAO* Fatty acid oxidation

#### RCT and cord blood

**Fig. 2:**
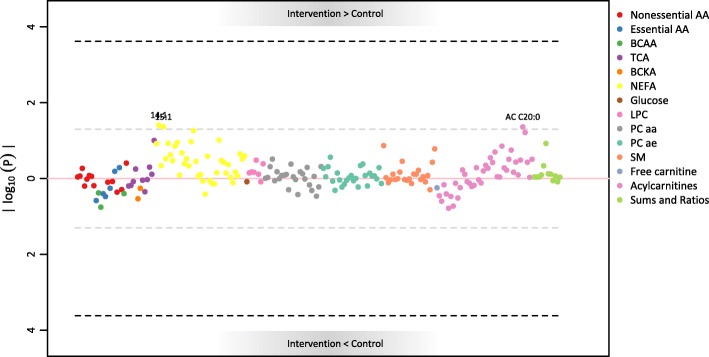
Manhattan plot for the association of cord metabolites with the RCT arm. Associations were calculated via generalized additive model with correction for maternal BMI, gestational age, foetal sex, sample storage time (non-linear effect) and random intercept for batch number. The full list of legend abbreviations is available in the methods section

No significant difference was found (see Fig. 2), and weak trends for elevated values in the intervention group were identified for only 3 metabolites: NEFA 14:1 and 15:1 and AC 20:0 (see Additional file 1). The subanalysis including only overlapping mother/child dyads delivered a similar picture, with PCaa 30:0, AC 4:0 and the branched-chain AA (BCAA) Valine (Val) showing a trend for lower values in the intervention group. Val, in particular, was more strongly different than the other two analytes (uncorrected *p* = 0.005). This subpopulation did not significantly differ in the baseline characteristics from the total population.

## Discussion

In this study, we explored the impact of a dietary intervention promoting a low-GI diet during pregnancy on the metabolome of pregnant women and cord blood of their offspring. We found that the low-GI diet was associated with consistently higher concentrations of phospholipids (PL) and acylcarnitines (AC) in maternal blood (though non-significant after correction for multiple testing), while cord blood metabolome was not substantially affected by the intervention.

### Maternal blood

All changes identified in maternal blood between control and intervention groups were related to fatty acids (FA), either non-esterified or in the form of acyl esters or PL chains. PLs are membranes lipids whose abundance is associated with both endogenous metabolism and dietary intake [14, 22]. The amount and composition of dietary fat consumed by the mothers did not change over pregnancy or due to the intervention, thus an exogenous change in fat intake can be excluded. It is possible that the low-GI diet modifies metabolism towards the release of fat from adipose tissue, hence the usage of fat as source of energy (as seen in the higher FAO markers and AC) and their transport (via PL).

A recent study by Hernandez-Alonso et al. found that a low-GI diet over 6 months was associated with changes in amino acid concentrations (both positively and negatively) and with a marked decrease in phospholipids, particularly SM and LPC, when compared both intra-subject to the patient’s own baseline levels or inter-subjects against patients following high GI or low fat diets [17]. In our results, subjects in the intervention group showed no differences regarding AA, but higher levels of phospholipids, especially those containing FA with 16 and 18 carbon atoms chains, than subjects in the control group. To evaluate these discrepancies, it should be noted that the populations and study design largely differed: Hernandez-Alonso et al. investigated overweight and obese men and women in a calories-restricted setting for over 6 months, while our population was composed of pregnant women in the second half of gestation who embraced an isocaloric, low-GI diet for 12 weeks. In particular, a 6 months calories-restricted diet can be seen as a prolonged catabolic state, while in pregnancy a first anabolic state spanning until the end of the first trimesters is followed by an accelerated catabolic state in the third trimester [19, 20, 29]; therefore, our data, collected at the end of the second trimester, might represent the peak of the anabolic phase or the begin of the catabolic phase.

All AA modulated by low-GI diet in Hernandez-Alonso’s study have been observed to decrease during gestation [29]; this progression has been linked to the increased placental uptake for foetal protein synthesis [29] and it is possible that the foetal needs might dominate over their regulation due to low-GI diet. Maternal phospholipids, especially PL and SM, have been observed to rise during pregnancy [29, 41]; this rise has been attributed to oestrogens [42] and similar differences in phospholipids have also been observed in young adult women taking hormonal contraceptives [38]. Moreover, a systematic review conducted by Goff et al. in 2013 found that low-GI diet reduces total and LDL cholesterol but does not affect HDL cholesterol [13] (meta-analysis estimate for reduction in LDL for a low-GI diet of 9–20 weeks from a total population of 1281 study subjects: − 0.16 mmol/l, 95% CI: − 0.24 to − 0.08). These results refer to intra-subject differences pre- and post-intervention. In our data, neither the absolute LDL concentrations at 28th week nor their difference to the baseline levels were different between the groups. During pregnancy, a marked increase in circulating lipoproteins occurs (e.g., LDL cholesterol is expected to increase from < 2.59 mmol/l in non-pregnant population to up to 5.8 mmol/l at the end of gestation) [1], as the high foetal demand for cholesterol is matched solely by maternal supply during the first two trimesters [18]. It is plausible that, also in the case of phospholipids, the cholesterol- and phospholipids-lowering effect of a low-GI diet might be inhibited by the major endocrinologic changes enacted to provide for the increased needs of mother and foetus.

Nevertheless, some of the species higher in the intervention group (total SM, SM C42:2 and C42:3) were found to be associated with LDL lipoproteins in pregnant women in a recent publication by Rauschert et al. [36]. Moreover, the absolute differences in GI in the population under investigation were very modest (see Table 2), so it is possible that a more intensive dietary intervention beginning pre-conceptually might in fact be beneficial for prospective mothers with potential lipid dysregulations.

As for how such changes are enacted, an interesting study in mice by Stavrovskaya et al. [40] found that GI and fat composition work synergistically in affecting the FA composition of cardiolipins, a subclass of mitochondrial PLs; that is, the changes in the FA composition of cardiolipids were more pronounced if the diet was high GI and high in trans or saturated fat than if either component was present alone. In our data, we could not test this hypothesis due to lack of variability in the dietary fat intake. Nevertheless, our results, combined with the findings from Stavrovskaya et al., urge further investigation of the mechanisms linking fat intake and GI to lipids composition and fat metabolism.

### Cord blood

Cord blood metabolome was largely unimpacted by the intervention. We did find a small difference in the concentration of Val between the RCT groups in the analysis of the overlapping subjects which might be indeed be ultimately linked to the lower maternal GI intake (BCAA levels correlate with and might cause insulin resistance [31], while a low GI diet should prevent it); however, since the finding was not replicated in the larger cohort and no other BCAA was different between the study groups, this result has to be interpreted carefully.

In general, data about the impact of lifestyle interventions on cord blood metabolome are scarce; however, our results from the ROLO cohort are in line with previous findings from the UPBEAT study [35], where a lifestyle intervention in pregnant women with obesity, while beneficial to the mothers, did not affect cord blood metabolome [34]. In particular, the enhanced availability of maternal lipids was not mirrored in higher transport to the foetus. This is not surprising, since placental FA transport is subject to complex regulatory mechanisms [5, 11, 39]. Nevertheless, there is the need of a deeper understanding of how maternal diet may influence placental transport, as the data to this regard are scarce [43].

Despite the ROLO trial found no effect of the intervention on birth and early infancy anthropometry measures [46], epidemiological studies show that the effects of in-utero exposures on metabolic health might become evident later in life [24, 26], e.g. because modulated via epigenetic changes [8, 28]. “Subtle but widespread” changes in the DNA methylation were found in the cord blood of babies from the ROLO study, as reported by Geraghty et al. [9]. In other words, the intervention might have not impacted foetal metabolism and early infancy anthropometry, but the in-utero exposure to a low-GI diet might still show its beneficial effects in later stages in life.

### Strengths and limitations

The strengths of our analysis were the large panel of metabolites (268 analytes, sums and ratios) studied with the same LC-MS/MS targeted approach, the availability of data from both maternal and cord samples, and the extensive dietary data. A major limitation of our study was the small sample sizes, which nevertheless did not prevent from inspecting the trends in maternal and cord blood. The small magnitude of the difference in GI between the study group and the lack of hormonal measurements as confounding factors might also have obscured additional differences and associations from being identified.

## Conclusions

Our analysis showed that a low-GI dietary intervention in pregnancy was associated with modest but consistent increases in maternal plasma phospholipids and utilization of fat as source of fuel, while cord blood was not affected by this intervention. Our study was the first to investigate the effect of a low-GI diet in a pregnant population. Our results were partially in agreement with studies conducted on non-pregnant subjects, and we ascribe discrepancies in the findings to the pregnancy-specific metabolic adaptations enacted to ensure sufficient nutrients to the developing foetus, for which more research is needed. Our exploratory findings may be used to direct further investigations about low-GI diets before and during pregnancy, to improve patient care for pre-conceptional and pregnant women with lipid dysregulations and potentially modulate the offspring’s risk for future onset of metabolic diseases.

## Additional files

Additional file 1: Results of the generalized additive models. (XLSX 83 kb)

Additional file 2: Sensitivity analyses for maternal blood. (DOCX 25 kb)

## Data Availability

The datasets during and/or analysed during the current study available from the corresponding author on reasonable request.

## Abbreviations

AA: Amino acids
AC: Acylcarnitine
BMI: Body-mass index
CV: Coefficient of variation
FA: Fatty acid
FAO: Fatty acid oxidation
GDM: Gestational diabetes mellitus
GI: Glycaemic index
GWG: Gestational weight gain
ISD: Internal standard
LC: Liquid chromatography
LPC: Lysophosphatidylcholine
MS: Mass spectrometry
NEFA: Non-esterified fatty acids
PCaa: Diacyl-phosphatidylcholine
PCae: Acyl-alkyl-phosphatidylcholine
PL: Phospholipids
QC: Quality control
RCT: Randomized controlled trial
SM: Sphingomyelin
