# Supplementary material for: Effect of a low glycaemic index diet during pregnancy on maternal and cord blood metabolomic profiles: results from the ROLO randomized controlled trial
Source: Nutr Metab (Lond). 2019 Aug 27;16:59. doi: 10.1186/s12986-019-0378-z (PMC6712779; doi:10.1186/s12986-019-0378-z)
Supplement: Supplementary file 2 — Sensitivity analyses for maternal blood. (DOCX 25 kb) [file 12986_2019_378_MOESM2_ESM.docx]

Additional file 2 to

Effect of a low glycaemic index diet during pregnancy on maternal and cord blood metabolomic profiles: results from the ROLO randomized controlled trial

Linda Marchioro^1^, Aisling A. Geraghty^2^, Olaf Uhl^1^, Engy Shokry^1^, Eileen C. O’Brien^2^, Berthold Koletzko^1^*, Fionnuala M. McAuliffe^2^

^1^ Division of Metabolic and Nutritional Medicine, Department of Paediatrics, Dr. von Hauner Children’s Hospital, University hospital, LMU Munich, Munich, Germany

^2^ UCD Perinatal Research Centre, School of Medicine, University College Dublin, National Maternity Hospital, Dublin, Ireland

*Supplement 1*: Results of the generalized additive models (spreadsheet).

*Supplement 2*: Sensitivity analyses for maternal blood (text file).

*Univariate sensitivity analyses*

*Methods.*

As univariate sensitivity analyses, we wanted to test the robustness of the results when correcting for GWG, fasting glucose, total cholesterol, HDL and LDL measured at 28^th^ week. Due to the small sample size, this could not be done on the main model. Therefore, we created a “metabolites residuals dataset” by computing the following GAM models and saving the model residual s(s(∙) indicates a non-linear effect, 1|∙ indicates random intercept):

*metabolite at 28 weeks ~ maternal BMI + metabolite at 13 weeks +*

*s(sample storage time) + 1|batch number*

We then used these metabolites residuals as dependent variables in linear regressions with RCT and one covariate (GWG, fasting glucose, total cholesterol, HDL and LDL measured at 28^th^ week ) as predictors.

*Results*

The results did not differ from those of the main models when adjusting for blood lipids or fasting glucose; however, when adjusting for GWG, most associations disappeared. Since, however, values for GWG were missing in 14 subjects (27% of the analysed data), we additionally repeated the basic analysis (univariate regression of metabolites residuals on RCT group belongingness) using only subjects with GWG data. In this repeated analysis, both models with and without correction for GWG showed the same analytes, namely PCae (28:2, 32:2 and 36:0), 4 SM (42:2, 42:3, 43:2 and 43:3), the marker of FAO 18:1, one NEFA (24:1) and one LPC (18:0 with alkyl-bond), to be higher in intervention than control group at uncorrected alpha-level of 0.05. We could not establish phenotypic differences in the subjects with or without GWG values, except for the absolute and proportional intake of saturated fat in trimester 2 (higher in the subject *with* GWG values than in those with missing values).

*Conclusion.*

Due to these results, we conclude that GWG, fasting glucose and blood lipid do not play a strong mediating role in the associations of maternal metabolites with the intervention.

*Multivariate sensitivity analyses*

*Methods.*

As multivariate sensitivity analyses, we applied random forests (RF) (with two implementations, via R packages randomForest (1) and varSelRF (2, 3)) and logistic regression with covariates selection (LASSO and elastic net, implemented in R package glmnet (4)). Since these methods require complete data at comparable scales, we used multiple imputation via chained equations (R package mice (5)) to impute the missing data in the residuals dataset (see univariate sensitivity analysis).

*Results.*

The following analytes were identified as being associated with the intervention in any multivariate approach (results from the univariate analysis for comparison):

| Univariate | RF (randomForest) | RF (varSelRF) | LASSO | Elastic Net |
| --- | --- | --- | --- | --- |
|  | Leu | Leu |  |  |
| NEFA 18:1 | NEFA 18:1 | NEFA 18:1 |  |  |
| PC ae C28:2 |  |  | PC ae C28:2 | PC ae C28:2 |
| PC ae C32:2 | PC ae C32:2 | PC ae C32:2 | PC ae C32:2 | PC ae C32:2 |
| SM C41:2 |  |  |  | SM C41:2 |
| SM C42:2 | SM C42:2 | SM C42:2 |  | SM C42:2 |
| SM C42:3 | SM C42:3 | SM C42:3 |  |  |
| SM C43:2 |  |  |  | SM C43:2 |
| SM C43:3 |  |  | SM C43:3 | SM C43:3 |
| AC C14:2 | AC C14:2 | AC C14:2 |  |  |

All analyses identified PCae 32:2 as being associated with the intervention, but the other identified metabolites differed. Leu was identified by the RF, though it was not significant in the univariate analyses. All methods had a high error rate (OOB for random forest: 47%, accuracy for logistic regression with variables selection: 46%).

*Conclusion.*

Due to these results, we conclude that the multivariate methods failed to identify a metabolomic profile strongly associated with the intervention.

*Dietary fat intake analysis*

*Methods.*

We have inspected the Spearman correlations of the 43 maternal analytes associated with RCT with the dietary fat intakes of trimester 1 and 2 (16 variables: Total fat, saturated, monounsaturated and polyunsaturated fat, both as absolute intake (g) and as percentage of total calories (%kcal), for trimesters 1 and 2), thus 43 * 16 = 688 univariate comparisons.

*Results.*

Without correction for multiple testing, only 10 of them (1.45%) were significant at 0.05 alpha level, and none of them was significant after false discovery rate (FDR) or Bonferroni correction. Three correlation were in magnitude larger than 0.35, all other correlations had smaller magnitudes.

| Analyte | Fat intake | r | p | p (FDR) | p (Bonferroni) |
| --- | --- | --- | --- | --- | --- |
| AC C10:0 | Polyunsaturated fat intake T2 (g) | -0.31 | 0.047 | 0.987 | 1 |
| LPC a C18:1 | Monounsaturated fat intake T1 (g) | 0.34 | 0.026 | 0.987 | 1 |
| PC aa C36:0 | Saturated fat intake T2 (g) | -0.44 | 0.003 | 0.987 | 1 |
| PC aa C36:0 | Saturated fat intake T2 (%kcal) | -0.36 | 0.017 | 0.987 | 1 |
| PC aa C36:0 | Total fat intake T2 (g) | -0.34 | 0.028 | 0.987 | 1 |
| PC ae C28:2 | Saturated fat intake T2 (%kcal) | -0.39 | 0.011 | 0.987 | 1 |
| PC ae C40:2 | Polyunsaturated fat intake T2 (%kcal) | 0.33 | 0.033 | 0.987 | 1 |
| SM C42:3 | Polyunsaturated fat intake T2 (%kcal) | 0.33 | 0.029 | 0.987 | 1 |
| SM C43:2 | Polyunsaturated fat intake T1 (g) | 0.33 | 0.035 | 0.987 | 1 |
| SM C35:2 | Total fat intake T1 (g) | 0.31 | 0.049 | 0.987 | 1 |

Except for PC aa C36:0, which seems to be (surprisingly) negatively correlated with total and especially saturated fat intake, all other analytes only appear once in this table.

*Conclusion.*

Due to these results, we conclude that the contribution of dietary fat is irrelevant to the metabolome differences associated with the intervention.

**References**

1. Liaw AW, M. Classification and Regression by randomForest. R News. 2002;2(3):18-22.

2. Diaz-Uriarte R. GeneSrF and varSelRF: a web-based tool and R package for gene selection and classification using random forest. BMC Bioinformatics. 2007;8(1):328.

3. Díaz-Uriarte R, Alvarez de Andrés S. Gene selection and classification of microarray data using random forest. BMC Bioinformatics. 2006;7(1):3.

4. Friedman JH, Hastie T, Tibshirani R. Regularization Paths for Generalized Linear Models via Coordinate Descent. 2010. 2010;33(1):22 %J Journal of Statistical Software.

5. van Buuren S, Groothuis-Oudshoorn K. mice: Multivariate Imputation by Chained Equations in R. 2011. 2011;45(3):67 %J Journal of Statistical Software.
